# xHMMER3x2: Utilizing HMMER3’s speed and HMMER2’s sensitivity and specificity in the glocal alignment mode for improved large-scale protein domain annotation

**DOI:** 10.1186/s13062-016-0163-0

**Published:** 2016-11-29

**Authors:** Choon-Kong Yap, Birgit Eisenhaber, Frank Eisenhaber, Wing-Cheong Wong

**Affiliations:** 1 Bioinformatics Institute (BII), Agency for Science, Technology and Research (A*STAR), 30 Biopolis Street, #07-01, Matrix, Singapore, 138671 Singapore; 2 School of Computer Engineering (SCE), Nanyang Technological University (NTU), 50 Nanyang Drive, Singapore, 637553 Singapore

**Keywords:** Sequence homology, Hidden Markov model, Sequence similarity search, Fold-critical sequence segment, Non-globular sequence segment, Similarity score dissection

## Abstract

**Background:**

While the local-mode HMMER3 is notable for its massive speed improvement, the slower glocal-mode HMMER2 is more exact for domain annotation by enforcing full domain-to-sequence alignments. Since a unit of domain necessarily implies a unit of function, local-mode HMMER3 alone remains insufficient for precise function annotation tasks. In addition, the incomparable E-values for the same domain model by different HMMER builds create difficulty when checking for domain annotation consistency on a large-scale basis.

**Results:**

In this work, both the speed of HMMER3 and glocal-mode alignment of HMMER2 are combined within the xHMMER3x2 framework for tackling the large-scale domain annotation task. Briefly, HMMER3 is utilized for initial domain detection so that HMMER2 can subsequently perform the glocal-mode, sequence-to-full-domain alignments for the detected HMMER3 hits. An E-value calibration procedure is required to ensure that the search space by HMMER2 is sufficiently replicated by HMMER3. We find that the latter is straightforwardly possible for ~80% of the models in the Pfam domain library (release 29). However in the case of the remaining ~20% of HMMER3 domain models, the respective HMMER2 counterparts are more sensitive. Thus, HMMER3 searches alone are insufficient to ensure sensitivity and a HMMER2-based search needs to be initiated. When tested on the set of UniProt human sequences, xHMMER3x2 can be configured to be between 7× and 201× faster than HMMER2, but with descending domain detection sensitivity from 99.8 to 95.7% with respect to HMMER2 alone; HMMER3’s sensitivity was 95.7%. At extremes, xHMMER3x2 is either the slow glocal-mode HMMER2 or the fast HMMER3 with glocal-mode. Finally, the E-values to false-positive rates (FPR) mapping by xHMMER3x2 allows E-values of different model builds to be compared, so that any annotation discrepancies in a large-scale annotation exercise can be flagged for further examination by dissectHMMER.

**Conclusion:**

The xHMMER3x2 workflow allows large-scale domain annotation speed to be drastically improved over HMMER2 without compromising for domain-detection with regard to sensitivity and sequence-to-domain alignment incompleteness. The xHMMER3x2 code and its webserver (for Pfam release 27, 28 and 29) are freely available at http://xhmmer3x2.bii.a-star.edu.sg/.

**Reviewers:**

Reviewed by Thomas Dandekar, L. Aravind, Oliviero Carugo and Shamil Sunyaev. For the full reviews, please go to the Reviewers’ comments section.

**Electronic supplementary material:**

The online version of this article (doi:10.1186/s13062-016-0163-0) contains supplementary material, which is available to authorized users.

## Author summary

Over the past decade, the OMICs frenzy from arrays to sequencing has swarmed genomic research with voluminous amount of data and elucidated lists of candidate genes/proteins. Yet, many of these genes/proteins remained not well-understood in relation to the observed phenotype. The major gap to our full understanding stems from our lack in complete gene/protein functions which, in turn, impedes researchers from assembling the sets of biomolecular mechanisms that can sufficiently explain the observed phenotype in these OMICs experiments; the knowledge of gene/protein functions is a premise necessary for delivering the big promises in personalized medicine.

Despite so, experimental characterization of gene/protein function still receives insufficient attention nowadays. This can be attested by the dwindling number of characterized genes/proteins reported over the past decade. Sadly, one can expect this number to continue to grow at a slow rate.

On this basis, the only viable approach is to computationally transfer function annotation of the well-studied gene/protein sequences to the less-studied or novel ones for functional hints. Implicitly, this entails that these in-silico function annotations must always be kept consistent regardless of the revisions made to function annotation databases or associated software tools used during the computations.

## Background

The value of biomedical and biotechnological applications from biomolecular sequence information is generally limited by the degree of functional annotation of non-coding genomic regions, protein-coding genes and the proteins themselves [1, 2]. Whereas experimental characterization of all genes in all organisms is out of question by their sheer number, computational annotation transfer from a smaller number of well-studied proteins greatly expands the space of functionally characterized protein sequences [3–5].

While lexical analysis and certain simple sequence motif-function correlations are helpful for understanding functions for non-globular protein segments, the homology concept based on significant levels of sequence similarity (mostly with regard to the hydrophobic pattern and functional residues indicative for conservation of fold, structure and function) is the foundation for understanding functions of globular protein regions [3–5]. A pair of sequences is considered homologous as a result of a statistical assessment of their alignment with a statistical mutation model quantified with an E-value criterion.

Since an element of chance implies a chance for error, unjustified annotations can arise and percolate into databases [6–9]. Thus, functional annotation is a challenging and non-trivial task [9–15].

Traditionally, large-scale domain function annotation is achieved through the HMMER [16–18] algorithm with companion domain libraries like Pfam [19, 20] and SMART [21, 22]. This involves searching against a library of functional domains for each query protein sequence, a process that needs to be repeated for all protein isoforms (many thousands even for bacterial genomes and typically dozens of thousands for a eukaryote genome) and for all genomes under study. The HMMER2 version with its glocal mode is highly valued by the genomics community for its exact domain placement over query sequences; i.e., it enforces full alignments to domain models (each domain being a unit of function); the latter being the modus operandi of the sequence homology concept. Additionally, HMMER2 is known for its reliability of E-value guided domain assignments despite slow computation. Fortunately, the recent HMMER3 is marked by its quantum leap in computation speed that makes any large-scale domain annotation task practical on standard computers. However, HMMER3 cannot reproduce HMMER2’s glocal mode alignments, it works just in the fragmented domain alignment mode. Hence, neither HMMER variants alone is a complete solution for practical large-scale annotation of known globular domain given the large stream of new genomic data from the current era.

Taken together, the preceding conditions give rise to the utility and design rationale behind xHMMER3x2. First, the basic idea is to utilize HMMER3’s improved speed to first scan for statistically significant sequence-to-domain hits. Then, the sequence-to-full-domain alignments of these hits are reproduced through the glocal-mode of HMMER2. Overall, this approach will greatly reduce the search space for HMMER2 so that xHMMER3x2’s overall speed will be most similar to HMMER3 while retaining the more desirable glocal-mode alignments. The major aspect is then to ensure that the search space of glocal-mode HMMER2 is sufficiently replicated by HMMER3 local-mode for any given E-value cutoff as faithfully as possible. We achieve this goal through the receiver operating characteristic (ROC) calibration of the domain library to be used by xHMMER3x2 so that the E-values for every domain model from different builds are made comparable through the false-positive rate measure.

In hindsight, the application of the calibration procedure on the Pfam domain library reveals three post-calibration findings that underline some of the fundamental differences between HMMER3’s search style and HMMER2’s glocal mode that justify for the necessity of xHMMER3x2 to unify both HMMER variants into a common framework. Another important outcome is that the false-positive quantification by xHMMER3x2 results in a key step for flagging discrepancies in large-scale domain annotation for manual scrutiny.

## Results and discussion

### Post-calibration finding 1: On average, HMMER3 E-values need to be more stringent than HMMER2 glocal-mode E-values to exhibit the same FPR

The function annotation task via the HMMER algorithm is innately coupled to the domain libraries. In this work, the Pfam library (release 29) with a total of 16295 domain models was examined. Furthermore, to capture the same search space of glocal-mode HMMER2 via the local-mode HMMER3, a calibration (or normalization) procedure is needed to relate the different E-values generated by either tool for the same sequences and domain models (See Methods on “Calibration procedure ..”).

Briefly, this entails the objective comparison of the receiver operating characteristic (ROC) curves generated by the two HMMER builds (i.e., HMMER2 and HMMER3) for each domain model. The query sequence set is specific to every domain model and it is composed of a positive set of *α* protein sequences (from the seed sequences of the Pfam domain model of interest, actually the potentially “true hits”) and the consensus sequences of the remaining 16294 domain models (if these sequences are hit, they are regarded as “false hits”).

**Fig. 1:**
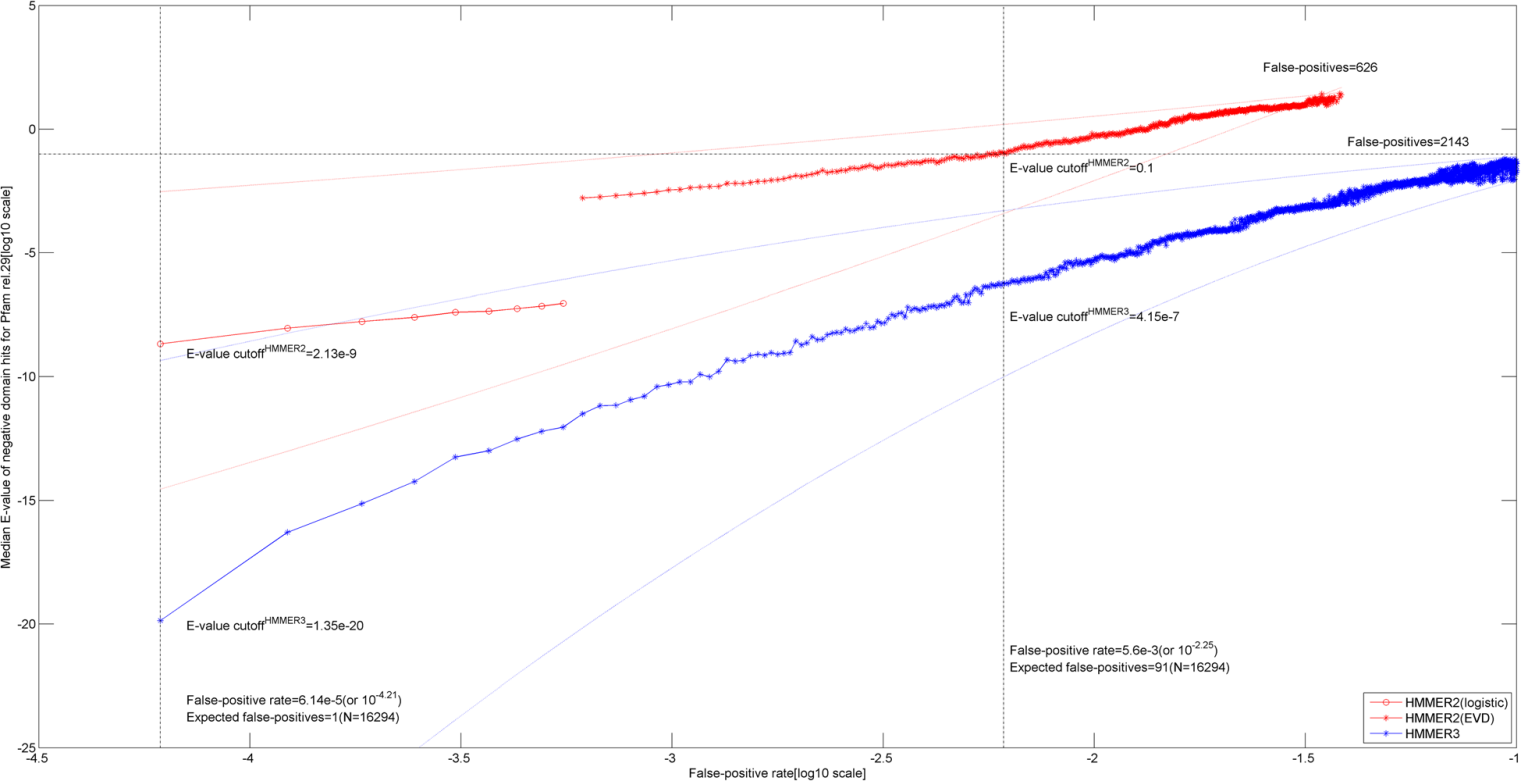
False-positive rates against median domain E-value cutoffs of the Pfam library (release 29) for HMMER2 and HMMER3 model builds. The HMMER2 (*in solid red*) and HMMER3 (*in solid blue*) plots are depicted with their IQRs (interquartile ranges) in dotted red and blue lines respectively. The average E-value cutoff of HMMER3 needs to be more stringent than HMMER2. The magnitude difference between them is about 10^6^ ~ 10^11^. When one imposes the recommended E-value cutoff of 0.1 for HMMER2, the corresponding FPR will be at 5.60e-3 (see dotted vertical line) with 91 false-positives. The equivalent HMMER3 E-value will be 4.15e-7. For extreme stringency (i.e., FPR < 6.14e-5 or FP < 1), the required HMMER2 and HMMER3 E-value cutoffs are 2.13e-9 and at 1.35e-20 respectively (*see leftmost dotted vertical line*)

Figure 1 depicts the false-positive rate (FPR) value against the median E-value of negative domain hits in Pfam library (release 29) for the two HMMER builds in a doubly logarithmic plot. The HMMER2 (in solid red) and HMMER3 (in solid blue) plots are shown with their interquartile ranges (IQRs) in dotted red and blue lines respectively. In a nutshell, the FPRs for each domain model are computed over 16294 negative domain consensus sequences of the Pfam library. For each domain, a list of *i* negative E-values are sorted in ascending order and labelled with its order from 1 to *i*. The order is in fact the FP value and the FPR range for this domain is, hence, between 1/16294 and *i*/16294.

Over the full body of the Pfam library, we found in our test calculations that the false-positive (FP) values span between 1 and 626 for HMMER2 and between 1 and 2143 for HMMER3. In turn, this translates to the sampled FPR ranges of between 6.14e-5 (1 out of 16294) and 3.84-2 (626 out of 16294) for HMMER2 and between 6.14e-5 and 1.32e-1 (2143 out of 16294) for HMMER3. Then, at each sample FPR or FP points, the median E-values of the negative domain hits of the Pfam library were taken to give the median E-value ranges of between 2.13e-9 to 2.40e + 1 for HMMER2 and between 1.35e-20 and 7.1e-2 for HMMER3. A reproducible feature to note for HMMER2 software suite is the occurrence of a breakpoint due to the switch in statistical model from the extreme value distribution (EVD) to the logistic model when the similarity score grows larger. In our previous work, the median E-value of Pfam domain (release 23) for this switch occurs at 1.0e-7 [10] which falls within the current (release 29) E-value breakpoint range of between 10^−3^ and 10^−8^.

Separately, the upper FPR limit of HMMER2 is lower than that of HMMER3 since it detects much less false-positive hits than HMMER3. This is unsurprising since HMMER2 enforces complete sequence-to-domain alignment when operating in glocal-mode, while HMMER3 does not (and the occurrence of a sequence similar to a fraction of the domain suffices). And for the intended purpose of capturing the search space by HMMER2, the common upper limit of FPR at 5.60e-3 (see label corresponding to 91 false-positives in Fig. 1) is theoretically sufficient for both HMMER variants, albeit at different E-value of 0.1 for HMMER2 and at 4.15e-7 for HMMER3.

In any case, an overall comparison of the two negative domain hits plots reveals that the median E-value needs to be more stringent in HMMER3 than in HMMER2 given a fixed FPR. The magnitude difference is about (10^6^ ~ 10^11^) across the sampled range. This is consistent with the HMMER manual’s recommended E-value cutoff of < <1 for HMMER3 and <0.1 (between 0.1 and 1 requires manual checking) for HMMER2 to gather trusted hits. As an example, when one imposes the recommended trusted domain E-value cutoff of 0.1 for HMMER2, the corresponding FPR will be at 5.60e-3 (see dotted vertical line). The latter translates into 91 false-positive hits. Meanwhile, the equivalent HMMER3 E-value will be 4.15e-7 at this FPR setting. If extreme stringency is applied to achieve zero FP (i.e., FPR <6.14e-5 or FP <1 given our search database size), then the required HMMER2 and HMMER3 E-values will be at 2.13e-9 and at 1.35e-20 respectively (see leftmost dotted vertical line in Fig. 1).

However, complicated situations can arise as the HMMER2 and HMMER3 E-values enter the lower FPR of below 5.6e-3 (FP = 91) where the IQR of the two plots start to overlap. Essentially, this implies that there can be cases where the HMMER2 E-values can exhibit higher stringency than its HMMER3 equivalent, therefore bucking the general trend of HMMER3’s stringency over HMMER2. As such, a direct comparison of E-values from different HMMER builds even for comparable sequence-to-domain alignments is not always a straightforward exercise.

### Post-calibration finding 2: About 20% of HMMER2 domain models have better sensitivity than the HMMER3 counterpart over all the FPR range

After the domain library calibration procedure, the HMMER builds with the better sensitivity can be evaluated for the domain library. Briefly, the area under curve (AUC; see Eq. 1) of the HMMER2 and HMMER3 models are first computed for a fixed FP value, *x*. These evaluated FP values, which correspond to respective FPRs (in parentheses), are *x*_1_ = 1 (6.14e-5), *x*_2_ = 5 (3.07e-4), *x*_3_ = 10 (6.14e-4), *x*_4_ = 20 (1.20e-3), *x*_5_ = 30 (1.80e-3), *x*_6_ = 40 (2.50e-3), *x*_7_ = 50 (3.10e-3), *x*_8_ = 60 (3.70e-3), *x*_9_ = 70 (4.30e-3), *x*_10_ = 80 (4.90e-3), *x*_11_ = 90 (5.50e-3) and *x*_12_ = 100 (6.14e-3). Furthermore, to enhance the stability of the model selection, a predefined set of FP values *A* (see Eq. 2) and its associated model counts (Eqs. 3 and 4) are evaluated and taken as the overall criteria for model selection (See Methods on “ Criteria for domain model build…” for details).

**Fig. 2:**
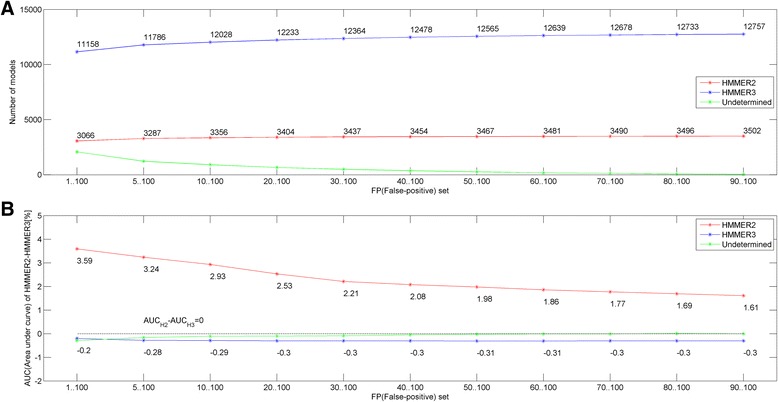
No. of more sensitive HMMER2 models and sensitivity performance of HMMER2 and HMMER3 Pfam (release 27) model builds. **a** depicts the proportions of HMMER2 (*red*), HMMER3 (*blue*) and undetermined (*green*) model builds for the various sets of FP values. Based on the HMMER2 plot (*in red*), a stringent set of 3066 to 3502 HMMER2 models were found to be more sensitive than their HMMER3 counterparts at all evaluated FP values. This is 21.5% of the evaluated Pfam domain library (release 27). On the other hand, 68.5% (11158/16295) to 78.3% (12757/16295) of the HMMER3 domain models remain more sensitive than their HMMER2 counterparts (*blue plot*). Up to 12.7% of the domain library fluctuates between HMMER2 and HMMER3 depending on the evaluated FP value (*green plot*). **b** shows the average normalized AUC difference (in terms of percentage) for the sets of HMMER2, HMMER3 and undetermined models. The average AUC difference ranges between 1.61 and 3.59% for HMMER2 models (*red plot*) while it is held steadily at about 0.3% (*blue plot*) for HMMER3. For the sets of undetermined models, the average AUC difference stabilizes to about 0%

Figure 2a depicts the total number (see Eqs. 5–7) of HMMER2 (in red), HMMER3 (in blue) and undetermined (in green) model builds selected by the various sets of FP. The FP set starts with all the evaluated FP values of 1 to 100. As the plot moves along the horizontal axis from left to right, the smaller FP values are dropped progressively. Based on the HMMER2 plot (in red), a stringent set of 3066 HMMER2 models were found to be more sensitive than their HMMER3 counterparts for all evaluated FP sets. As the smaller FP values are dropped, more HMMER2 domains were added until a maximum of 3502 models have been reached. This is about 21.5% of the evaluated Pfam domain library (release 29). Meanwhile, a large proportion of HMMER3 domain models remain more sensitive than their HMMER2 counterparts. Quantitatively speaking, this is between 68.5% (11158/16295) and 78.3% (12757/16295) of the domain library (blue plot). A sizeable percentage of domain models (up to 12.7% of the Pfam library) has shown to be fluctuating between HMMER2 and HMMER3 depending on the evaluated FP value set (green plot) and hence, not deterministic for selection.

Figure 2b shows the normalized AUC difference (see Eq. 1) being expressed as percentage and averaged over the sets of selected HMMER2/HMMER3/undetermined domain models corresponding to Fig. 2a. Basically, a large difference implies higher sensitivity of either the HMMER2 or HMMER3 models. Specifically for the HMMER2 sets (red plot), the average AUC difference ranges between 1.61 and 3.59% while this is held steadily at about 0.3% (blue plot) for the HMMER3 sets. For the sets of undetermined models, the average AUC difference stabilizes to 0% as more low FP values were excluded (green plot) and are hence deemed to perform closer to the HMMER3 models.

Overall, the domain sensitivity evaluation revealed that about 20% (between 3066 and 3502; see Additional file 1 from xHMMER3x2 website) of the HMMER3 models were found unsuitable for the initial domain scanning in the xHMMER3x2 workflow. Interestingly, these 3502 models were biased towards shorter domain models with a median of 171 alignment positions (IQR = 170). The latter is suggestive of alignment quality issues when coupled to the local-mode HMMER3. In any case, these HMMER3 models cannot capture the HMMER2 search space sufficiently and hence, their HMMER2 model counterparts need to be used despite a longer computational speed. Fortunately, the slowdown caused by this 20% of the Pfam library is not detrimental; the slowest xHMMER3x2 will still outperform the standalone glocal-mode HMMER2 at least by an order of magnitude (see below, Results section 5).

In hindsight, larger numbers of domain hits (which can include false-positives at the same time) can generally be expected from local-style search mode (as in HMMER3) compared with the glocal mode regime (as in HMMER2) since alignments over the full domain model are not enforced in the first case. Fortunately, HMMER3 provides larger number of hits and, as a trend, better sensitivity than HMMER2. However, on the flip side, HMMER3’s tendency towards partial domain coverage is insufficient to justify for function annotation transfer since a full domain necessarily implies a unit of function. The extent of HMMER3’s partial coverage over various FPR values is quantified in the next section.

### Post-calibration finding 3: Average domain coverage by HMMER3 positive hits is about 88% of the domain model length

In a strict sense, partial sequence-to-domain alignments generally do not justify for a sequence segment having the function of the domain hit since the completeness of a 3D structural unit (as implied by the domain unit) is a precondition for functional annotation transfer. The latter is especially relevant to HMMER3 since it can only operate in local-mode.

**Fig. 3:**
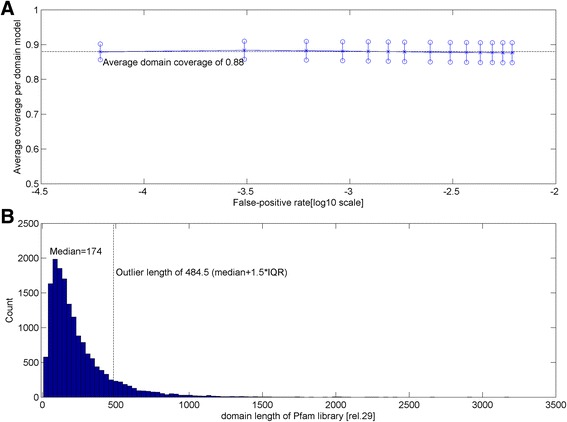
Average coverage per domain model and average True-positive rate of the Pfam domain models (release 27). In **a**, an average for each domain model is computed by taking the total coverage of detected positive hits divided by the total detected positive hits. The average of a full domain coverage is one. An overall average is taken over all 16295 HMMER3-build Pfam models for each false-positive rate (FPR). Across the FPR range, the average domain coverage of the Pfam domain models hovers about 0.88 as indicated by the horizontal dotted line. **b** gives the distribution of domain length of the Pfam library. There are a total of 1801 domains with length of 485 alignment positions or more (i.e., larger than 1.5 times the inter-quartile range above the median; see vertical line in **b**)

To study the completeness of domain coverage by HMMER3, we first computed the average “coverage per domain model” by taking the total coverage of detected positive hits divided by the total detected positive hits at a FPR for a given domain model. Finally, an overall average is taken over all 16295 HMMER3-build Pfam models. Figure 3a depicts across the sampled FPR range of between 6.14e-5 and 6.14e-3 (in logarithmic scale) together with the standard error bars at each evaluated FPR value. Briefly, the “coverage per domain model” gives an idea of how much the sequence-to-domain alignment hits cover the domain model on average. If the domain coverage of a model is complete in all cases, then the average is expected to be one. Across the FPR range, the average domain coverage of the Pfam domain models with HMMER3 straddles at about 0.88 (see horizontal dotted line) with the lower limit (median-1.5IQR) of the average domain coverage at about 0.85.

Although the average “coverage per domain model” remains relatively stable across all FPR values, incomplete domain coverage is expected to have different impact especially in cases of relatively long and of the shorter domain models. Figure 3b gives the distribution of domain length of the Pfam release 29 library. There are a total of 1801 domains with length of 485 alignment positions or more (i.e., >median + 1.5IQR; see vertical line in Fig. 3b). Therefore, the lack of coverage constitutes at least 58 ((1–0.88) × 484.5) missing alignment positions. The latter is about the size of the smallest known domain (e.g., zinc finger models); thus, completeness of the respective domain range in the query sequence is at least doubtful. On the other hand, there are 1429 with domains length of 58 alignment positions and less, where annotation transfers are even more unjustified under incomplete coverage. Taken together, these 3230 (1801 + 1429) implicated models make up almost 20% of the Pfam library. Therefore, despite HMMER3’s generally superior sensitivity, it suffices only for initial fast domain detection. Ultimately, HMMER2 will still be required for re-alignment over the full domain model, especially for the shorter ones.

### The xHMMER3x2 workflow and webserver implementation

**Fig. 4:**
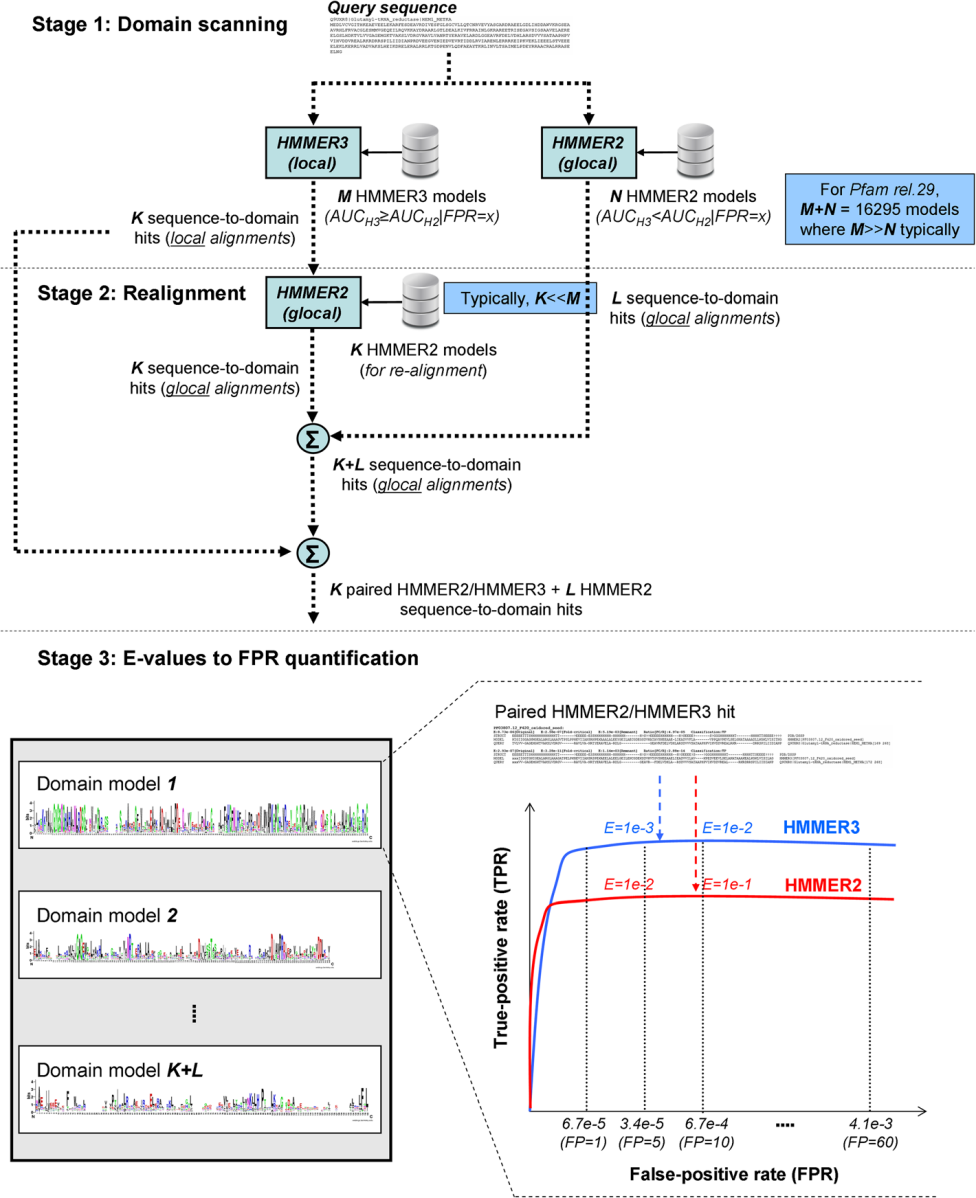
The xHMMER3x2 webserver workflow. The three-staged xHMMER3x2 workflow is shown. In the first stage of xHMMER3x2, *N* (3066 ~ 3502) HMMER2 and *M* (13229 ~ 12793) HMMER3 models can be utilized for the search (where *N < <M*) based on the sensitivity evaluation between HMMER2 and HMMER3 models in Fig. 2a. Upon the model selection, the respective hmmpfam (HMMER2) and hmmscan (HMMER3) runs are executed with the respective sub-libraries of HMMER2 and HMMER3 domain models. In the second stage, the two runs will result in *K* local-mode and *L* glocal-mode sequence-to-domain alignments respectively. The resulting K local-mode alignments will be searched against the same *K* domain models but in glocal-mode HMMER2 to perform the full domain re-alignment step. The third stage of xHMMER3x2 estimates the FPR(FP) values of the domain hits from their E-values using the previously calibrated HMMER2/HMMER3 ROCs. The FPR intervals are bounded by the FPR(FP) points: 6.14e-5 (1), 3.07e-4 (5), 6.14e-4 (10), 9.21e-4 (15), 1.20e-3 (20), 1.50e-3 (25), 1.80e-3 (30), 2.50e-3 (40), 3.10e-3 (50), 3.70e-3 (60), 4.30e-3 (70), 4.90e-3 (80), 5.50e-3 (90) and 6.14e-3 (100)

Figure 4 gives an overview of the three-staged xHMMER3x2 workflow. Briefly, xHMMER3x2 first invokes HMMER3 to scan for initial domain hits over a set of query sequences that matches the search space of HMMER2. The hits are then carried over to HMMER2 for refinement into full sequence-to-domain alignments.

In the first stage of xHMMER3x2, *N* (3066 ~ 3502) HMMER2 and *M* (13229 ~ 12793) HMMER3 models can be utilized for the search (where *N < <M*) based on the sensitivity evaluation between HMMER2 and HMMER3 models in Fig. 2a. The upper E-value limit is set to 0.1 for HMMER3 and 24 for HMMER2 which corresponds to the maximum sampled FP values of 2143 and 626 respectively according to Fig. 1. Essentially, the smaller search space of HMMER2 should be sufficiently captured.

Generally speaking, since the total number of utilized HMMER3 models far exceeds that of HMMER2 (80% versus 20%), the speed of xHMMER3x2 is expected to be closer to the faster HMMER3 than the slower glocal-mode HMMER2. As such, reasonable computational speed can be achieved without sacrificing for detection sensitivity but with the caveat that the HMMER3 hits might be fragmented to various extents. Upon the selection of the appropriate model builds, the respective hmmpfam (HMMER2) and hmmscan (HMMER3) runs are executed with the respective sub-libraries of HMMER2 and HMMER3 domain models.

In the second stage, the two runs from the dominant HMMER3 and the minority HMMER2 will result in *K* local-mode and *L* glocal-mode sequence-to-domain alignments respectively for a given query sequence. The resulting K local-mode alignments will be searched against the same *K* domain models but in glocal-mode HMMER2. The latter is considered as the full domain re-alignment step as intended by glocal-mode HMMER2 originally. Since the number of models used in the realignment step is expected to be much less than the total number of HMMER3 models used (i.e., *K < <N* < <*M*), this is an area of major speed gain by xHMMER3x2 over pure HMMER2. At the end of stage two, xHMMER3x2 would have produced *K* pairs of HMMER2/HMMER3 and *L* HMMER2 sequence-to-domain hits over *K + L* domain models for every query sequence.

The third stage of xHMMER3x2 estimates the FPR (and FPs out of 16294) values of the domain hits based on their E-values. For each domain model, the previously calibrated HMMER2/HMMER3 ROCs are purposed to assign the particular domain hits to the appropriate FPR interval based on their E-values. The intervals are bounded by the evaluated FPR (FP) points: 6.14e-5 (1), 3.07e-4 (5), 6.14e-4 (10), 9.21e-4 (15), 1.20e-3 (20), 1.50e-3 (25), 1.80e-3 (30), 2.50e-3 (40), 3.10e-3 (50), 3.70e-3 (60), 4.30e-3 (70), 4.90e-3 (80), 5.50e-3 (90) and 6.14e-3 (100) as depicted in Fig. 4. The estimated FPR (FP) values of the domain hits are simply the nearest margin of their assigned FPR interval.

The overall xHMMER3x2 workflow is implemented as a software suite in PERL language. It is freely available for download at http://xhmmer3x2.bii.a-star.edu.sg for local installation. Since xHMMER3x2 requires the carefully-selected domain models for its proper performance, the list of calibrated models for the recent Pfam domain libraries (release 27, 28 and 29) are also provided at the same site. Alternatively, an online webserver (for up to 1000 FASTA-formatted sequences) exists at the download site as well. When multiple sequences are inputted, the result is returned via email in tabulated form as electronically readable file containing query sequences, Pfam model hits together with their alignments E-values and standardized FPRs. Upon input of a single sequence, the HMMER2 and HMMER3 results are presented graphically-enhanced for easy comparison.

### xHMMER3x2 improves on computational speed over HMMER2 and domain-detection sensitivity over HMMER3

To evaluate the real-world performance of xHMMER3x2, the sequences and Pfam domain annotations of the human UniProt/SwissProt sequences were evaluated. The source file was extracted from the public resource at ftp://ftp.uniprot.org/pub/databases/uniprot/current_release/knowledgebase/complete/uniprot_sprot.dat.gz.

To create a reference set for evaluation purpose, the sequences were re-annotated via the glocal-mode HMMER2 against the Pfam domain library (release 29). Only the re-annotated domains that overlap with the existing UniProt/SwissProt Pfam annotations were retained. The latter aims to exclude UniProt annotations that cannot be reproduced by this computation as a result of direct inheritance from previous releases, percolation errors, etc. Overall, our re-annotation resulted in 27878 (out of 27988 reported annotations) Pfam domain hits which span across 17517 sequences over 5719 unique domain models. Since the complete human proteome is a moving scientific target [23], the re-annotated set used here is only for benchmarking purpose only. The list of originally-reported and re-computed domain annotations for the human UniProt/SwissProt sequences is available at the xHMMER3x2 website (see Additional file 2). Furthermore, a cutoff E-value of 1e-9 (corresponds to the negative domain E-value for FP < 1 in Fig. 1) was applied to derive a stringent set of 24696 positives domains which now covers 16175 sequences over 5509 unique domain models.

To examine the trade-offs between computational speed and domain-detection sensitivity, these 16175 sequences were subjected to hmmscan/hmmpfam searches via HMMER3 (as reference), glocal-mode HMMER2 and 5 configurations of xHMMER3x2 (where *N = 3502, N = top 1500/1000/500 and N = 0)* against the Pfam library (release 29). The top most sensitive HMMER2 domain models were ranked according to the AUC evaluation via Eq. 1.

**Fig. 5:**
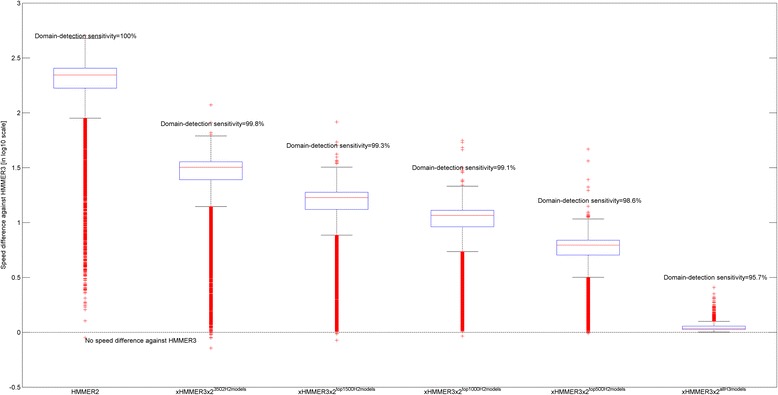
Domain-detection sensitivity and speed differences of HMMER2 and xHMMER3x2 against HMMER3. The leftmost boxplot represents the baseline difference where the average speed of HMMER2 is about 222× slower than HMMER3. However, the sensitivity of HMMER2 is 4.3% (100% versus 95.7%) higher than that of HMMER3. The rightmost boxplot represents the fastest xHMMER3x2 by running all HMMER3 models where its sensitivity is equal to that of HMMER3 at 95.7%. The trade-offs between computational speed and domain-detection sensitivity is given by the middle boxplots. As *N* (i.e., the number of default HMMER2 models) decreases from 3502,1500,1000 to 500 (see second to fifth boxplots from right), xHMMER3x2’s sensitivity decreases from 99.8, 99.3, 99.1 to 98.6% respectively. In contrast, its speed increases accordingly from 32×, 17×, 12× to 6.2× (*M*_*h*2_/(*M*_*h*3_ + *K*_*h*3_) = 10^0.81^) slower than HMMER3. Alternatively, this translates to 7×, 13×, 18.5× and 36× faster than HMMER2 respectively. The best compromise is achieved via the top 1000 most sensitive HMMER2 domains with the remaining as HMMER3 models, to attain a domain-sensitivity of 99.1% and a speed of 12× slower than HMMER3 but 18.5× faster than HMMER2

Figure 5 illustrates the domain-detection sensitivity and speed differences against HMMER3 (in log scale) for both HMMER2 and xHMMER3x2. The horizontal dotted line across the x-axis sets the reference where there is no speed difference from HMMER3. The leftmost boxplot depicts the most time-consuming HMMER2 at about 221 times (i.e., *M*_*h*2_/*M*_*h*3_ = 10^2.34^; see left-hand side of Eq. 12) slower than HMMER3, but with the improved sensitivity of 4.3% (100% versus 95.7%) over HMMER3. This translates to capturing 1074 more hits than HMMER3. In hindsight, this finding corroborates with our earlier post-calibration finding that some HMMER3 domain models have lower sensitivity than HMMER2.

For the various setting of xHMMER3x2, the fastest is achieved by running all HMMER3 models (i.e., *N = 0*; see rightmost boxplot) which is only 1.1 times (i.e., 10^0.04^) slower than HMMER3 (due to the necessary full domain re-alignment of glocal-mode HMMER2 in xHMMER3x2; see Fig. 4 Stage 2) but about 201 times (221÷1.1) faster than HMMER2. In hindsight, the re-alignment step of xHMMER3x2 does not impede its overall speed and the condition where *K < <N < <*M is generally true. However this is not without a caveat that its sensitivity has been compromised to 95.7% (same as HMMER3).

Meanwhile, the trade-offs between speed and sensitivity can be observed when the various sets of selected HMMER2 models were invoked by xHMMER3x2. Based on Fig. 5, as *N* (i.e., the number of default HMMER2 models) decreases from 3502,1500,1000 to 500 (see second to fifth boxplots from left), xHMMER3x2’s sensitivity decreases from 99.8, 99.3, 99.1 to 98.6% respectively. In contrast, its speed increases accordingly from 32× (*M*_*h*2_/(*M*_*h*3_ + *K*_*h*3_) = 10^1.50^; see right-hand side of Eq. 12), 17× (*M*_*h*2_/(*M*_*h*3_ + *K*_*h*3_) = 10^1.23^), 12× (*M*_*h*2_/(*M*_*h*3_ + *K*_*h*3_) = 10^1.07^) to 6× (*M*_*h*2_/(*M*_*h*3_ + *K*_*h*3_) = 10^0.80^) slower than HMMER3. Alternatively, this translates to 7 × (222÷32), 13 × (221÷17), 18.5 × (221÷12) and 37 × (221÷6.2) faster than HMMER2 respectively. Overall, the best compromise is achieved with the top 1000 most sensitive HMMER2 domains while the remaining as HMMER3 models. For most occasions, this configuration is the recommended and default mode in the xHMMER3x2 webserver since it achieves the best balance between domain-sensitivity (at 99.1%) and speed (12× slower than HMMER3 but 18.5× faster than HMMER2). However, when fast annotation is the main consideration, xHMMER3x2 offers the “glocal-mode HMMER3” as its fastest mode that is almost as fast as the native local-mode HMMER3.

In summary, the xHMMER3x2 framework will always produce the glocal-mode alignments and it can be configured to achieve a balance between speed and domain-detection sensitivity. On one extreme, it can be configured for full speed when all HMMER3 models are selected, thus mimicking a ‘glocal-mode’ HMMER3. On the other end, when all HMMER2 models are deployed, xHMMER3x2 reverts back to HMMER2 with the highest level of domain-detection sensitivity.

### False-positive quantification of calibrated domain hits allows for direct comparison between HMMER variants to evaluate for annotation consistency

At the end stage of xHMMER3x2, the FPR (FP) values for all the sequence-to-domain hits would have been estimated accordingly. To evaluate the consistency of domain annotation, the 23622 common domain hits to both HMMER2 and HMMER3 from the preceding analyzed set of 16175 human UniProt/SwissProt sequences were further examined.

**Fig. 6:**
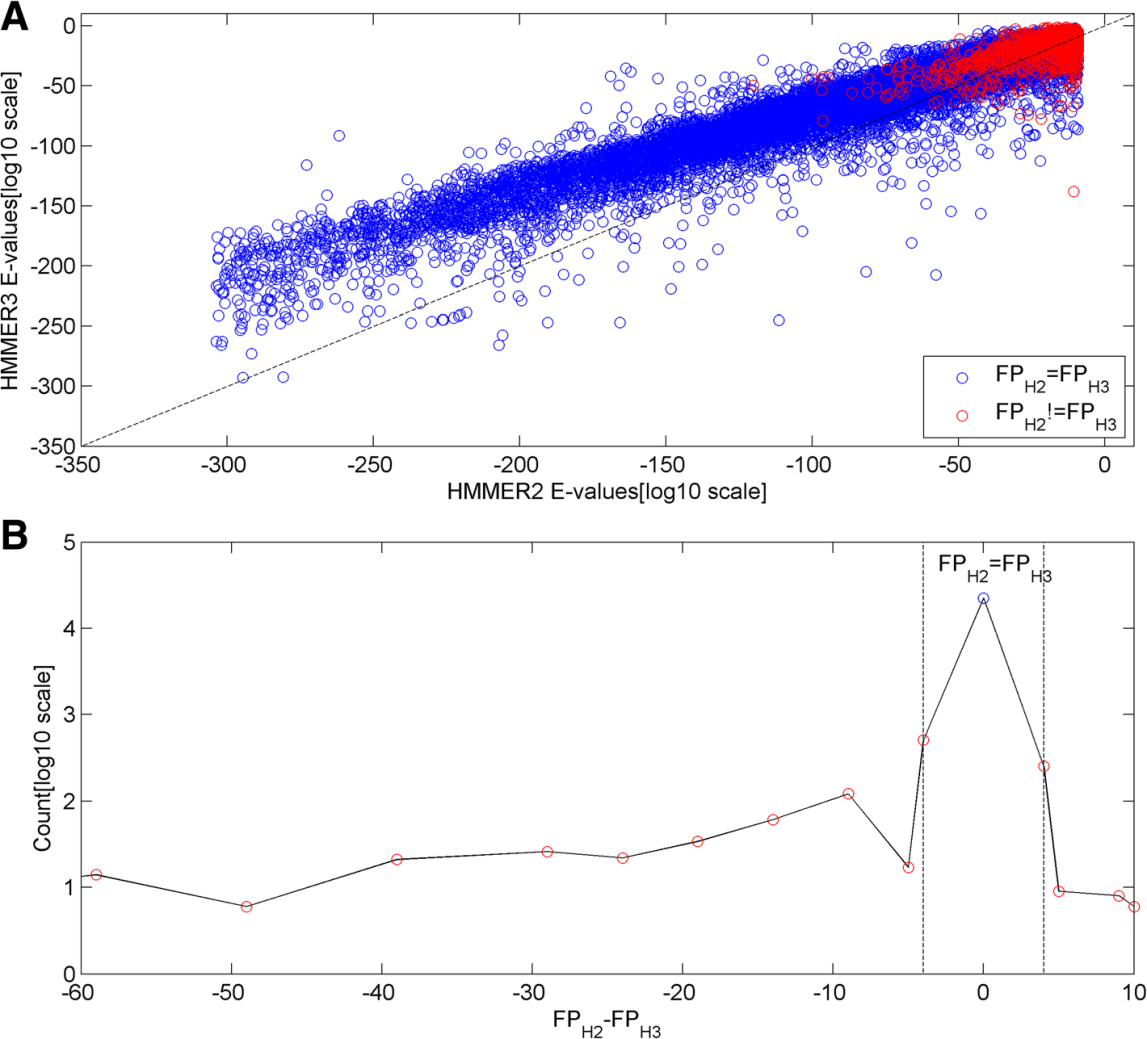
HMMER2/HMMER3 E-value (in log_10_ scale) plot and false-positive difference histogram of the UniProt/SwissProt human sequence domain hits. **a** depicts 23622 pairs of HMMER2/HMMER3 E-values where the blue points denotes cases where the estimated FP values of the pair matched, while the red points denote the unmatched cases. About 86.5% of the pairs show higher stringency of HMMER2 E-values over HMMER3, and vice versa for the remaining pairs. **b** shows the histogram of the 23622 pairs of FP difference (i.e., FP_H2_-FP_H3_) values of the paired HMMER2/HMMER3 hits. Basically, 95% of the data points converge to the singular point at FP_H2_ = FP_H3_ or 0 (*see blue dot*). If small estimates of FP_H2_-FP_H3_ = ±4 (*see vertical dotted lines*) are included, the overall concordance of the HMMER pairs is about 98% of the data points. - The left skewness implies that a handful 389 data pairs exhibits higher HMMER3 FPR than that of HMMER2. These pairs are highlighted for annotation inconsistency as a result of FPR quantification by xHMMER3x2

We define annotation consistency as the concordance in statistical assessment of same domain hits between HMMER2 and HMMER3 for a particular sequence segment. Figure 6a depicts the 23622 pairs of HMMER2/HMMER3 E-values (as log-log plot) that now covers 5492 unique domain models. The set of blue points denotes the case where the estimated FP values of the paired HMMER2/HMMER3 hits matched exactly, while the red points denote the unmatched cases.

Based on the linear regression analysis, the mathematical relationship of HMMER2 against HMMER3 E-values was found to be *y = 0.72x, R* ^*2*^ *= 0.91* (matched cases) and *y = 0.77x, R* ^*2*^ *= 0.22* (unmatched cases). In other words, the paired HMMER2/HMMER3 E-values are not equivalent, and non-linear to each other particularly for the unmatched cases given the bad *R* ^*2*^ (coefficient of determination) values. Additionally, about 86.5% of the pairs show higher stringency of HMMER2 E-values over HMMER3, and vice versa for the remaining pairs. To reiterate, direct comparison using E-values to determine concordance proves to be difficult. It is also important to remember that the evaluated Pfam annotations in UniProt are generally constrained to one domain hit per sequence segment. In more realistic settings, the same sequence segment can have multiple significant domain hits. In the absence of FPR estimation, the actual error rates associated to the overlapping domain hits will simply be masked behind a set of inseparable E-values.

Utilizing the estimated FP values of the paired HMMER2/HMMER3 hits, the difference (i.e., FP_H2_-FP_H3_) values were taken. The histogram of the 23622 pairs of FP difference values were shown in Fig. 6b. In contrast to the E-value plot, 95% (22428) of the data points converges to the singular point at FP_H2_ = FP_H3_ or 0 (see blue dot). When the small difference of FP_H2_-FP_H3_ = ±4 (see vertical dotted lines) are also considered since FP values are approximated to their nearest values, the overall statistical concordance of the HMMER pairs makes up about 98% (addition of another 766) of the data points. As such, FP quantification by xHMMER3x2 offers an easy and quick way to check for general concordance of the paired HMMER2/HMMER3 hits.

Unique to xHMMER3x2, its FPR quantification helps to highlight an annotation inconsistency based on an observed left-skewness in the histogram. More specifically, a handful ([FP_H2_-FP_H3_ < −4] = 389) data pairs exhibited higher HMMER3 FPR than that of HMMER2 despite similar sequence-to-domain alignments. These 389 outliers, which span across 140 unique Pfam domain models (see Additional file 3 from xHMMER3x2 website). Once again, the problematic domain models belong to the shorter ones; this set of 140 domains has a median length of 166.5 alignment positions (IQR = 204).

To resolve the annotation discrepancies, the sequences containing the 389 domain hits were subjected to a dissectHMMER [13–15, 24] analysis where pairs of HMMER E-values are dissected into their fold-critical and remnant contributions. Briefly, it is the fold-critical parts (the supposedly 3D structural part) of a sequence-to-domain alignment that argues for a similar overall fold, hence similar function whereas the remnant part represents disordered, fibrillary or other non-globular regions that might be not obligatory for the domain fold [13, 14, 24]. As such, the E-values of the fold-critical contributions has to be more significant than the remnant parts to justify for annotation transfer.

**Fig. 7:**
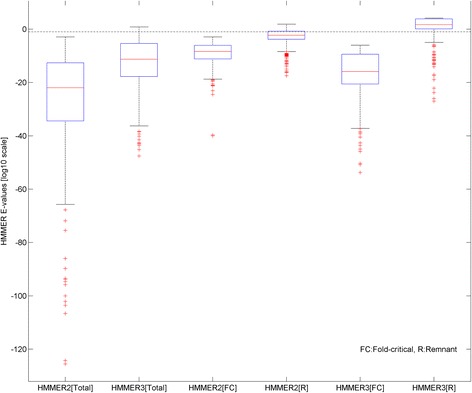
Boxplots of total, fold-critical and remnant E-values of the unmatched 389 domain hits from UniProt/SwissProt human sequences. The baseline for significance is marked by the dotted horizontal line of -1. The first two boxplots from the left shows that the median HMMER2 E-values are more significant than that of HMMER3. But when the fold-critical E-values of the pair data are considered (*see centre two boxplots*), the opposite is observed. In hindsight, the opposite trend is attributed to the heavily penalized remnant E-values of HMMER3 (*last two from left*) which causes the total HMMER3 E-values to be lowered

Figure 7 shows the boxplots of the total, fold-critical and remnant HMMER2 and HMMER3 E-values of the 389 dissected domain hits. As a baseline for significance, the dotted horizontal line (at -1) denotes the HMMER2’s recommended E-value of 0.1. The first two boxplots on the left shows that the total HMMER2 E-values of the 389 unmatched cases displays higher significance than that of HMMER3. Meanwhile, the fold-critical E-value boxplots display higher significance than the remnant boxplots whether for the case of HMMER2 or HMMER3. Additionally, both the fold-critical boxplots were considered significant when compared to their corresponding remnant counterparts. Taken together, the dissectHMMER analysis is strongly suggestive that these 389 HMMER2/HMMER3-based Pfam annotations were justifiable by their fold-critical plots; the annotation discrepancy is resolved.

Scrutiny of the remnant boxplots further revealed that the remnant E-values of the HMMER3 were so heavily penalized that they exceeded the recommended cutoff of 0.1 (−1 in log10 scale). Therefore, when the scores were added up as part of the total HMMER3 E-values, the latter becomes much less significant than that of HMMER2 and hence their larger FPRs. It is likely that HMMER3 cannot justify any independent non-globular alignment segments of the domain model without significant score compensation from other alignment pieces. Unfortunately, it is beyond the scope of this work to examine why the remnant segments of these particular HMMER3 models appeared to be so heavily penalized though this sheds some light on why certain HMMER3 models were less sensitive than their HMMER2 counterparts.

## Conclusion

Protein domain function annotation for the experimentally-unverified ones is at best a computational prediction that is based on the statistical evaluation of the sequence homology concept. With every new revision of any domain model library or the associated software tool, the corresponding annotation update becomes a frequent and routine task; the task of ensuring annotation consistency with every software or library migration is rarely cared for. Simultaneously, as more new genomes are being churned out, the need for a reliable and fast protein annotation workflow, that takes into account of annotation consistency, cannot be ignored.

In this work, the proposed xHMMER3x2 annotation framework is squarely aimed at alleviating both annotation discrepancies and speed issues that can arise from practical large-scale protein annotations. Notably, its scalability will allow for the future inclusion of newer HMMER variant. It simply requires the calibration of this new variant over a common domain library to allow its E-values to be compared against the others for selecting the most sensitive variant-dependent domain model amongst all. The remaining task is then to carefully balanced for the annotation speed.

## Methods

### Calibration procedure for HMMER2 and HMMER3 domain models towards comparable false-positive rates

The function annotation task via the HMMER algorithm is innately coupled to the domain libraries. The calibration procedure used here is generally applicable to any domain libraries to normalize between the two different model builds. It aims to capture the search space of glocal-mode HMMER2 via the local-mode HMMER3, therefore relating the different E-values generated by the two HMMER variants. In a nutshell, we determined the true-positive rate (i.e., sensitivity) and false-positive rate (i.e., 1-specificity) for the two HMMER builds (i.e., HMMER2 and HMMER3) of any domain model.

**Fig. 8:**
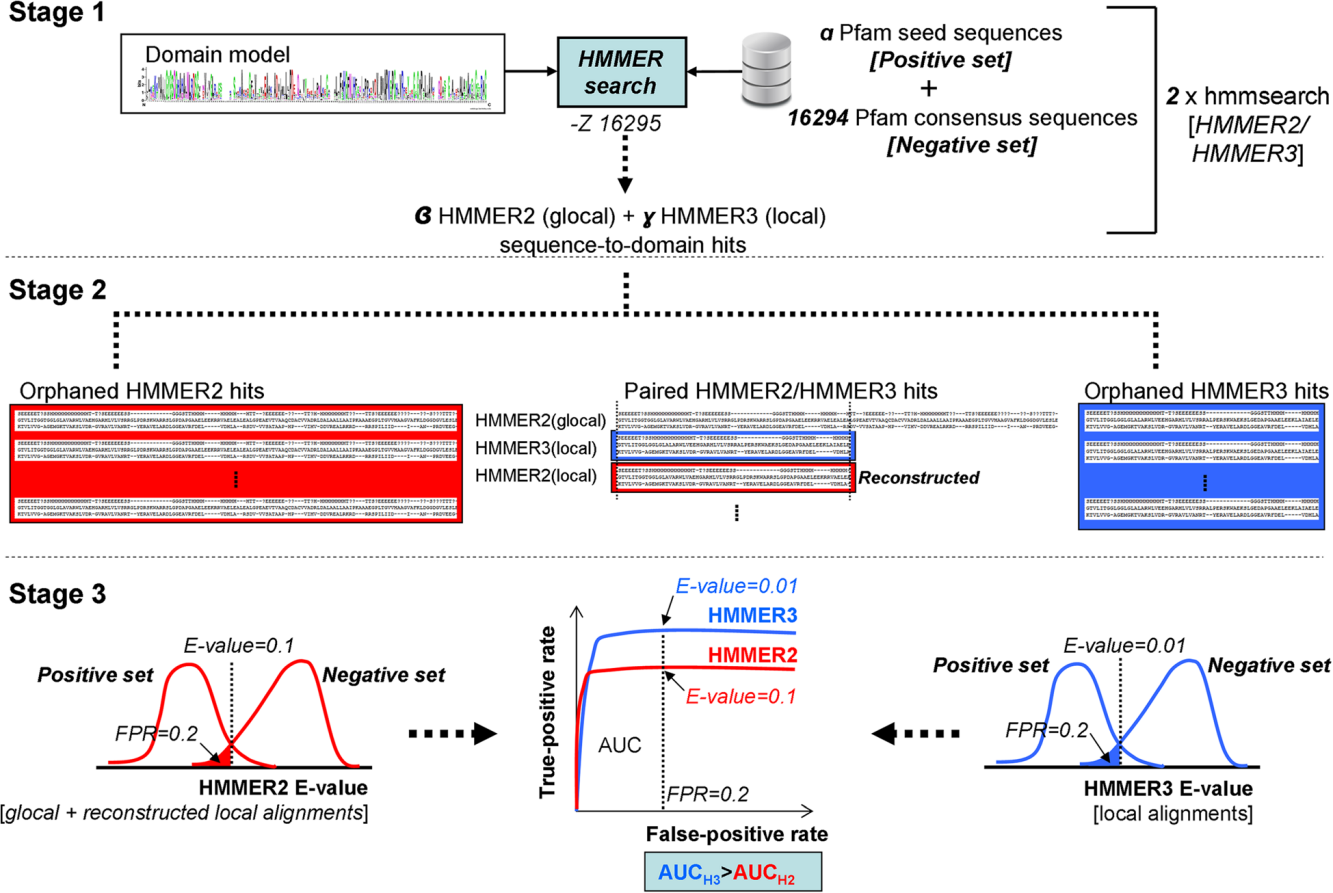
Calibration procedure between HMMER2 and HMMER3 domain model builds. Stage 1: The “hmmsearch” results for the two HMMER variants are first produced on a positive set of *α* seed sequences of the model with the rest of the 16294 models as consensus sequences. Stage 2: A total of *β* glocal HMMER2 and *γ* local HMMER3 sequence-to-domain alignments are paired HMMER2/HMMER3 hits or orphaned accordingly. For the paired hits, the HMMER2 hit is reconstructed to mimic the local alignment of its HMMER3 counterpart to derived a new HMMER2 E-value. Stage 3: the orphaned and reconstructed E-values of the HMMER2 are partitioned into their positive and negative sets (*see red histograms*). The same is done for the HMMER3 (*see blue histograms*). For a particular E-value cutoff, the respective false-positive rates (FPR) and true-positive (TPR) rates can be quantified from the two sets of histograms

The calibration workflow is schematically shown in Fig. 8. In stage 1 of Fig. 8, the “hmmsearch” sequence-to-domain alignments and corresponding E-values of the two HMMER variants are first produced over a query set of *α* + 16294 sequences for every domain model. The query sequence set is specific to every domain model and it is composed of a positive set of *α* protein sequences (from the seed sequences of the Pfam domain model of interest, actually the potentially “true hits”) and the consensus sequences of the remaining 16294 domain models (if these sequences are hit, they are regarded as “false hits”). The query sequences are extracted from the Pfam domain library alignments and are downloaded from ftp://ftp.ebi.ac.uk/pub/databases/Pfam/releases/Pfam29.0/Pfam-A.full.gz.

Aside applying the E-values cutoff of 1000 to capture as many hits as possible, the E-values were also calculated for the database size of 16295 (via the -z option) instead of *α* + 16294, so that the E-values will corroborate with hmmpfam(HMMER2)/hmmscan(HMMER3) search results in the subsequent xHMMER3x2 workflow. At the end of stage 1, two separate computations would have been executed and produced *β* HMMER2 and *γ* HMMER3 sequence-to-domain alignments over the space of *α* + 16294 query sequences.

In stage 2 of Fig. 8, the *β* glocal HMMER2 and *γ* local HMMER3 sequence-to-domain hits can be stratified into paired HMMER2/HMMER3 hits, HMMER2-only hits and HMMER3-only hits. More specifically, the paired hits are HMMER2 and HMMER3 hits where the domain model sequences overlap (see dissectHMMER for details [13, 14]). The orphaned HMMER2 and HMMER3 hits are those that are found only by the HMMER variant itself. For the paired HMMER2/HMMER3 hits, each glocal-mode HMMER2 hit is reconstructed to reproduce the local alignment made by its HMMER3 counterpart. As a result, a new E-value for this reconstructed local-mode HMMER2 alignment is also derived following published procedures [9, 13, 14].

In stage 3 of Fig. 8, the orphaned glocal-mode and reconstructed local-mode E-values of HMMER2 are partitioned into their positive and negative sets (see red histograms). The same is done for the paired and orphaned HMMER3 E-values (see blue histograms). For each designated E-value cutoff, the respective false-positive rates (FPR) and true-positive rates (TPR) can be quantified from the two sets of histograms. Consequently, the ROC (receiver operator curves) of HMMER2 (in red) and HMMER3 (in blue) can be overlaid on each other and made comparable at any FPR value. As an example, HMMER2 and HMMER3 require E-value cutoffs of 0.1 and 0.01 respectively to achieve a false-positive rate of 0.2 for the particular domain model used for this diagram. Also, the resulting AUC (area under curve) for HMMER3 is larger than that for HMMER2 at FPR = 0.2 based on the criteria (see blue box) and, hence, indicates better sensitivity.

It is important to note that marginally large FPRs or certain higher E-values can nevertheless suggest correct yet distant hits rather than totally wrong ones. Thus, our computed FPRs reflect the worst case thresholds. For a domain library like Pfam with clan classification of families of related domains, this is especially true.

### Criteria for domain model build selection

The base criteria for isolating a higher HMMER2 sensitivity at a particular FPR can be written into the conditional statement:

1 $$ {\overline{AUC}}_{diff}\left|{}_{FP=x}=\frac{AU{C}_{H2}- AU{C}_{H3}}{x/16295}>0\right|{}_{FP=x} $$

where *AUC*_*diff*_ is the difference between the area under curves (AUC) of HMMER2 and HMMER3, *x* is a fixed false positive rate. Here, the AUC value is normalized to total area under evaluation where 16294 is the total number of negative domains for any Pfam domain (release 29) model. Furthermore, to ensure that the sensitivity of an evaluated domain model does not fluctuate at different absolute numbers of false-positives (FPs), a counting mechanism is enforced to ensure that Eq. (1) holds over a set *A* which comprises of numbers of FPs or corresponding FPRs given as :

2 $$ A=\left\{{x}_1,{x}_2,\dots, {x}_n\right\} $$

while, the equation for the count based on (1) for domain model *y*, can be written as follows:

3 $$ coun{t}_y={\displaystyle \sum_{A=\left\{{x}_1,{x}_2,\dots, {x}_n\right\}}^{\left|A\right|}{\overline{AUC}}_{diff}} $$

where |A| is the cardinality of the set *A* of selected FPs of Eq. (2). As an example, a HMMER2 domain model is considered better than its HMMER3 alternate counterpart only when the following condition is satisfied :

4 $$ coun{t}_y=\left|A\right| $$

Conversely, a zero count or *count =* 0 describes the opposite case where the sensitivity of the HMMER3 model is better than that of HMMER2 at all FP sets.

In our study, the set of selected cases of false-positive hits (FP) corresponding to respective FPRs (in parentheses) are: *x*_1_ = 1 (6.14e-5), *x*_2_ = 5 (3.07e-4), *x*_3_ = 10 (6.14e-4), *x*_4_ = 20 (1.20e-3), *x*_5_ = 30 (1.80e-3), *x*_6_ = 40 (2.50e-3), *x*_7_ = 50 (3.10e-3), *x*_8_ = 60 (3.70e-3), *x*_9_ = 70 (4.30e-3), *x*_10_ = 80 (4.90e-3), *x*_11_ = 90 (5.50e-3) and *x*_12_ = 100 (6.14e-3).

Finally, the total number of selected HMMER2/HMMER3 and undetermined domain models for the set *A* of selected FP value, are as follows :

5 $$ tota{l}_{HMMER2}={\displaystyle \sum_{y=1}^{16295}\left\{\begin{array}{cc}\hfill coun{t}_y=\left|A\right|\hfill & \hfill 1\hfill \\ {}\hfill else\hfill & \hfill 0\hfill \end{array}\right.} $$

6 $$ tota{l}_{HMMER3}={\displaystyle \sum_{y=1}^{16295}\left\{\begin{array}{cc}\hfill 0< coun{t}_y<\left|A\right|\hfill & \hfill 1\hfill \\ {}\hfill else\hfill & \hfill 0\hfill \end{array}\right.} $$

7 $$ tota{l}_{\mathrm{undetermined}}={\displaystyle \sum_{y=1}^{16295}\left\{\begin{array}{cc}\hfill coun{t}_y=0\hfill & \hfill 1\hfill \\ {}\hfill else\hfill & \hfill 0\hfill \end{array}\right.} $$

### Theoretical speed improvement of xHMMER3x2 over glocal-mode HMMER2

Theoretically, the speed improvement of xHMMER3x2 over the glocal-mode HMMER2 algorithm can be quantified in the following manner. First, let the total run time of xHMMER3x2 for one sequence be defined as:

8 $$ {\displaystyle \sum_{i=1}^M{t}_{h3,i}}+{\displaystyle \sum_{k=1}^{K\subset M}{t}_{h2,k}}+{\displaystyle \sum_{i=M+1}^{M+N}{t}_{h2,i}} $$

Also, let the run time of HMMER2 be defined as:

9 $$ {\displaystyle \sum_{i=1}^M{t}_{h2,i}}+{\displaystyle \sum_{i=M+1}^{M+N}{t}_{h2,i}} $$

where *M*, *N* is the number of HMMER3 and HMMER2 models being used for a given FPR setting, *K* is the number of HMMER2 models used to re-align the HMMER3-derived sequence-to-domain alignments (i.e., *K* ⊂ *M*), *t*_*h*3,*i*_ and *t*_*h*2,*i*_ refer to the HMMER3 and HMMER2 computational time required for domain model *i*.

Against HMMER2, the speed improvement (expressed by a factor *f*) is attributed to the set of *M* models. Therefore, let the relevant terms in xHMMER3x2 (see Eq. 8) and HMMER2 (see Eq. 9) be related by the factor *f* via the following:

10 $$ f\left[{\displaystyle \sum_{i=1}^M{t}_{h3,i}}+{\displaystyle \sum_{k=1}^{K\subset M}{t}_{h2,k}}\right]={\displaystyle \sum_{i=1}^M{t}_{h2,i}} $$

Furthermore, let ${\displaystyle \sum_{i=1}^M{t}_{h3,i}}={M}_{h3}$, ${\displaystyle \sum_{k=1}^{K\subset M}{t}_{h2,k}}={K}_{h2}$ and ${\displaystyle \sum_{i=1}^M{t}_{h2,i}}={M}_{h2}$ where *t*_*h*3,*i*_ and *t*_*h*2,*i*_ are a unit of time in HMMER2 and HMMER3 respectively, then Eq. (10) can be simplified as *f*[*M*_*h*3_ + *K*_*h*2_] = *M*_*h*2_ and rearranged as follows :

11 $$ f=\frac{M_{h2}}{M_{h3}+{K}_{h2}} $$

where random variables *M*_*h3*_ and *M*_*h2*_ describe the total time taken for a sequence to perform hmmscan (HMMER3) and hmmpfam (HMMER2) searches respectively, against a database of *M* domain models (Stage 1 of Fig. 4). Similarly, random variable *K*_*h2*_ is the total time taken for a sequence to complete hmmpfam search against a set of *K* domain models (Stage 2 of Fig. 4).

Finally, the theoretical upper limit of xHMMER3x2’s speed improvement over HMMER2 is bounded by $\frac{M_{h2}}{M_{h3}}$ (i.e., HMMER2 versus HMMER3) as follows:

12 $$ \frac{M_{h2}}{M_{h3}}>\frac{M_{h2}}{M_{h3}+{K}_{h3}} $$

## Reviewers’ comments

### Reviewer’s report 1: Thomas Dandekar, Department of Bioinformatics, University of Wuerzburg, Germany

Choon-Kong Yap et al. make a good case that HMMER3 speed and HMMER2’s sensitivity and specificity can be well combined for improved large-scale protein annotation. All is made available for the interested reader, xHMMER3x2 is ready for use by the community.

There is not much to correct: the method is well explained and detailed, and, most of all, made fully available. Already the abstract explains that now the user has the choice from the script to go for faster annotation or higher accuracy (highest was 99.8%), fastest is HMMER3 glocal mode, but only 95.7% detection sensitivity. And again, all is well explained and as far as I can judge technically sound and well prepared I think what would now help to add, is a bit more practical experience (so simply explain in the discussion, what you already did do, did you for instance always take the “fast but sloppy” mode, so in essence HMMER3, or is there maybe a magical mix (such as BLOSUM62 with 62%) of HMMER2 optimal in your hands and in your experience? Finally, also comment how maybe future advances or other methods would influence this annotation procedure.

Authors’ response: *We thank the reviewer for his comments. We have added in the discussion to advise users to run xHMMER3x2 configured with the top 1000 HMMER2 domains as the recommended mode since this has the best balance in terms of sensitivity and speed. Also, the webserver now flags this configuration as the recommended mode under the mode selection list. The discussion on how xHMMER3x2 will be influenced by future advances or other methods is included in the conclusion.*

### Reviewer’s report 2: L Aravind, Protein and Genome Evolution Research Group, Computational Biology Branch, NCBI/NLM/NIH

HMMER3 has revolutionized HMM based search approaches by speeding up the searches greatly. However, this has come at the cost of preciseness of domain annotation that HMMER2’s glocal mode allowed. The authors attempt to remedy this compromise by combining the initial search with HMMER3 for detecting the domain followed by a HMMER2-like alignment procedure for the detected hits. Overall the glocal search procedure of HMMER2 does seem to provide more complete alignments which are often useful to ascertain domain boundaries. I have not compiled the program to run locally but testing on the server shows that it runs at comparable speed to a Pfam scan of single sequence with HMMER3.

I have no particular issues with the overall methodology. But I have some practical comments as a user who pays close attention to the quality of alignments: 1) Even though the technically the alignments produced by the web-server should be similar to a local HMMER2 Pfam scan, the examples I used produced “aesthetically” poorer alignments in that they were more gap-ridden. Is this arising from the attempt to cover the entire length of the Pfam model? 2) On trying a sequence where the match genuinely should not cover the entire Pfam model the alignments appear to be artificially stretched out to cover the entire length. While these issues might not greatly negatively affect an annotation pipeline for domains on a large-scale they might still affect the use of this search for in depth domain analysis in particular cases.

Authors’ response: *We thank the reviewer for his comments. For this work, all positions in the domain models’ seed multiple sequence alignments were considered during HMMER model building. On a large-scale basis, it helps to simplify the HMMER2* versus *HMMER3 sequence-to-domain hit comparisons, especially for cases where missing domain model positions can vary between HMMER2 and HMMER3 due to their parameterization differences.*

*At the same time, the quality of the sequence-to-domain alignment is also highly dependent on the curated seed alignments of the domain libraries. The visually poorer alignments as observed by the reviewer for the sequence example XP_002989768.1 was generated with Pfam release 29 for a pair of SBDS (PF01172) HMMER2/HMMER3 hits and a single SBDS_C (PF09377) HMMER2 hit. When the same example was tested on Pfam release 27, a stark improvement in alignment quality was made. Also, an additional SBDS_C (PF09377.5) HMMER3 domain hit, which covers about 2/3 of the model’s length, was also recovered. On closer examination of these alignments, we noted that the full model lengths of the SBDS and SBDS_C domains in Pfam releases 27 and 29 vary by 38 (132* versus *170) and 365 (234* versus *599) positions respectively. Mainly, the artificially stretched sequence-to-domain HMMER2 SBDS_C hit as observed with Pfam release 29, was due to a much longer domain model which includes more non-globular regions.*

*On a separate note, we wish to highlight that the seed alignments from Pfam release 28 and upwards were constructed from the reference proteome instead of the UniProt sequences. Given the same Pfam model, any differences in the seed sequences between releases will have a major impact on the final sequence-to-domain alignment quality. The implication of this sequence database change, in terms of search sensitivity/specificity, alignment quality,* etc. *will require a separate scrutiny.*

The search server could simplify the delivery of results by having the results directly presented rather than needing an additional click on the xHMMER3x2 query status page that is presented first.

Authors’ response: *The search server has now simplified the delivery of results by having the results directly presented rather than needing an additional click on the xHMMER3x2 query status page.*

### Reviewer’s report 3: Oliviero Carugo, Chemistry Department, University of Pavia

The manuscript submitted by Eisenhaber and Wong describes a novel computational approach for protein function annotation on the basis of sequence similarity. A web-server is also presented, to allow biologists to use this computational methodology. The basic idea is to couple two methods. One, HMMER2, is slow and the other, HMMER3, is fast. However, HMMER2’s glocal more alignments cannot be performed by HMMER3. Moreover, HMMER2 is probably more reliable than HMMER3, especially in the E-value-based domain assignments. The scientists lead by Eisenhaber and Wong designed an empirical interface, xHMMER3x2, which seems to be reliable and fast. Empirical parameters have been adapted based on PFAM-29.

The manuscript is very technical and sometime not easy to read. Most of the users of the server will probably never read the manuscript, which is too complex for the average biologist. However, it is important to publish a detailed description of this computational method, to allow bioinformaticians to understand it and, possibly, to improve it. On the contrary I warmly suggest the Authors to prepare a more colloquial description of this methods and to make it available in the web pages at http://xhmmer3x2.bii.a-star.edu.sg. This would greatly help biologists to try to understand the basics features of this method. About the computational procedure. Large use is made of the ROC curves. I have nothing again it. However, it is a better idea, in my opinion, to couple the ROC with the precision-recall curves (see for example a simple description published by [25]). Eventually, the Authors are required to read carefully the manuscript, which contains some minor mistakes. For example, in the Introduction (lines 13 and 14) one can read “A pair of sequences is considered homologues …” while it should be “homologous” (adjective). Another example, in reference 4 is written “Edited by Edited by”. In fine, the resolution of the figures seem insufficient to me.

Authors’ response: *We thank the reviewer for his comments. As suggested by the reviewer, a simplified description of our method is now available online and accessible* via *the “introduction” link right after the main title of the xHMMER3x2 webserver page. Alternatively, the link is given as* *http://xhmmer3x2.bii.a-star.edu.sg/introduction.html* *.*

*With regard to the use of ROC in our work, it is squarely aimed at calibrating between two different builds of the same domain mode to select for the more sensitivity one to be executed in xHMMER3x2. In fact, ROC was not used for performance measure. Rather, xHMMER3x2 was evaluated simultaneously for both speed and sensitivity. Nevertheless, the precision-recall (PR) curve helps to reveal any large class imbalance problem after the domain model calibration step. As such, the xHMMER3x2 webserver now presents a link to both the ROC and precision-recall (PR) curves of every domain model in the returned results.*

**Fig. 9:**
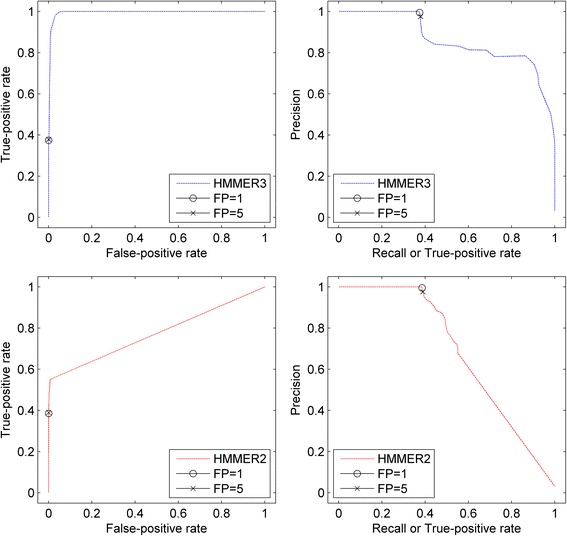
Receiver Operating Characteristics (ROC) and Precision-Recall (PR) curves of PF03915 AIP3 domain model. Depicts the ROC and PR curves (*HMMER3 in blue; HMMER2 in red*) for the domain model PF03915 AIP3. Based on the plot, at a FP of 1 and 5, the trusted AIP3 domain hits score a precision value of approximately 1.0 based on the PR curve and a true-positive/recall rate of about 0.4 based on the ROC curve. Beyond FP = 5, the precision rate and false-positive rate deteriorates quickly

*In hindsight, we are mindful of the limitations of any ratio measures (true-positive rates/recall rates, false-positive rates, precision rates) that may mask the true characteristics of the underlying data. Therefore, xHMMER3x2 reports the absolute number of false-positives (in discrete steps of FP = 1,5,10,15, …) that is associated to every sequence-to-domain alignment hit, much like how the E-values were intended for. Generally speaking, the FP range of 1 to 5 is relatively reliable for annotation purpose while any value beyond is considered for discovery purpose. As an example, Fig*. 9 *depicts the ROC and PR curves (HMMER3 in blue; HMMER2 in red) for the domain model PF03915 AIP3. From the figure, trusted AIP3 domain hits have the precision value of about 1.0 (see PR curve) with the true-positive/recall rate of about 0.4 (see ROC) as marked by FP = 1 and 5. Beyond that, the precision rate deteriorates with a synchronized increase in false-positive rate.*

### Reviewer’s report 4: Shamil Sunyaev, Division of Genetics, Dept. of Medicine, Brigham & Women’s Hospital and Harvard Medical School

The authors try to find a balance between two versions of HMMER (the more recent version offering a much greater speed at the price of slightly reduced sensitivity). They offer an umbrella approach combining both versions and adding estimation of false-positive rate. Although addressing a technical point, this work provides a potentially useful protein domain annotation software.

I find that the analysis of FPR should be clarified. The analysis suggests that E-values for both versions of HMMER are not perfectly tuned. Is this due to specific assumptions in the statistical model of HMMER, or this is a result of the choice of the negative dataset?

Authors’ response: *We thank the reviewer for his comments. Indeed, the reviewer’s first intuition is correct. The fundamental difference stems from the statistical models which gives rise to the different sets of HMMER2 and HMMER3 scoring parameters (*i.e.*, the emission and transition probabilities). As such, even comparable sequence-to-domain alignments can give quite different E-values. In addition, when comparing alignments between partial HMMER3 and full HMMER2 hits, only the overlapping segments were considered for re-deriving the HMMER2 scores. Despite so, these E-values are still not comparable.*

## Additional files

Additional file 1: List of top 1500/1000/500 better HMMER2 Pfam29 domain models. (TXT 76 kb)
Additional file 2: List of original vs re-computed Pfam domain annotations for the human UniProt/SwissProt sequences. (TXT 12216 kb)
Additional file 3: List of 140 Pfam domains with annotation inconsistencies. (TXT 10 kb)

## Abbreviations

FPR: False positive rate
HMM: Hidden Markov Model
IQR: Interquartile range
ROC: Receiver Operating Characteristics
TPR: True positive rate
